# Supplementary material for: A fault-intrusion-tolerant system and deadline-aware algorithm for scheduling scientific workflow in the cloud
Source: PeerJ Comput Sci. 2021 Nov 2;7:e747. doi: 10.7717/peerj-cs.747 (PMC8576554; doi:10.7717/peerj-cs.747)
Supplement: Supplemental Information 6 [file peerj-cs-07-747-s006.zip › Myproject1.1/docs/org/workflowsim/clustering/TaskSet.html]

TaskSet


JavaScript is disabled on your browser.


- Overview
- Package
- Class
- Use
- Tree
- Deprecated
- Index
- Help

- Prev Class
- Next Class

- Frames
- No Frames

- All Classes

- Summary:
- Nested |
- Field |
- Constr |
- Method

- Detail:
- Field |
- Constr |
- Method


org.workflowsim.clustering

## Class TaskSet

- java.lang.Object
- - org.workflowsim.clustering.TaskSet

- ---

    

  ```
  public class TaskSet
  extends java.lang.Object
  ```

  TaskSet is a group of tasks used only in clustering

  Since:
  :   WorkflowSim Toolkit 1.0

- - ### Field Summary

    Fields

    | Modifier and Type | Field and Description |
    | --- | --- |
    | `boolean` | `hasChecked` the check point. |
  - ### Constructor Summary

    Constructors

    Constructor and Description

    `TaskSet()`

    Initialize a TaskSet object
  - ### Method Summary

    Methods

    | Modifier and Type | Method and Description |
    | --- | --- |
    | `void` | `addTask(java.util.ArrayList<Task> list)` Adds a list of task to this taskSet |
    | `void` | `addTask(Task task)` Adds a task to this taskSet |
    | `java.util.ArrayList<TaskSet>` | `getChildList()` Gets the child list |
    | `double` | `getImpactFactor()` Gets the impact factor |
    | `long` | `getJobRuntime()` Gets the job runtime of this taskSet (sum of task runtime) |
    | `java.util.ArrayList<TaskSet>` | `getParentList()` Gets the parent list |
    | `java.util.ArrayList<Task>` | `getTaskList()` Gets task list |
    | `void` | `setImpactFafctor(double factor)` Sets the impact factor |

    - ### Methods inherited from class java.lang.Object

      `clone, equals, finalize, getClass, hashCode, notify, notifyAll, toString, wait, wait, wait`

- - ### Field Detail


    - #### hasChecked

      ```
      public boolean hasChecked
      ```

      the check point.
  - ### Constructor Detail


    - #### TaskSet

      ```
      public TaskSet()
      ```

      Initialize a TaskSet object
  - ### Method Detail


    - #### getImpactFactor

      ```
      public double getImpactFactor()
      ```

      Gets the impact factor

      Returns:
      :   impact factor


    - #### setImpactFafctor

      ```
      public void setImpactFafctor(double factor)
      ```

      Sets the impact factor

      Parameters:
      :   `factor` - the impact factor


    - #### getParentList

      ```
      public java.util.ArrayList<TaskSet> getParentList()
      ```

      Gets the parent list

      Returns:
      :   parent list


    - #### getChildList

      ```
      public java.util.ArrayList<TaskSet> getChildList()
      ```

      Gets the child list

      Returns:
      :   the child list


    - #### getTaskList

      ```
      public java.util.ArrayList<Task> getTaskList()
      ```

      Gets task list

      Returns:
      :   task list


    - #### addTask

      ```
      public void addTask(Task task)
      ```

      Adds a task to this taskSet

      Parameters:
      :   `task` - to be added


    - #### addTask

      ```
      public void addTask(java.util.ArrayList<Task> list)
      ```

      Adds a list of task to this taskSet

      Parameters:
      :   `list` - to be added


    - #### getJobRuntime

      ```
      public long getJobRuntime()
      ```

      Gets the job runtime of this taskSet (sum of task runtime)

      Returns:
      :   job runtime


- Overview
- Package
- Class
- Use
- Tree
- Deprecated
- Index
- Help

- Prev Class
- Next Class

- Frames
- No Frames

- All Classes

- Summary:
- Nested |
- Field |
- Constr |
- Method

- Detail:
- Field |
- Constr |
- Method
